# Autonomous Flying With Neuromorphic Sensing

**DOI:** 10.3389/fnins.2021.672161

**Published:** 2021-05-14

**Authors:** Patricia P. Parlevliet, Andrey Kanaev, Chou P. Hung, Andreas Schweiger, Frederick D. Gregory, Ryad Benosman, Guido C. H. E. de Croon, Yoram Gutfreund, Chung-Chuan Lo, Cynthia F. Moss

**Affiliations:** ^1^Central Research and Technology, Airbus, Munich, Germany; ^2^U.S. Office of Naval Research Global, London, United Kingdom; ^3^United States Army Research Laboratory, Aberdeen Proving Ground, Maryland, MD, United States; ^4^Airbus Defence and Space GmbH, Manching, Germany; ^5^U.S. Army Research Laboratory, London, United Kingdom; ^6^Department of Bioengineering, Imperial College London, London, United Kingdom; ^7^Institut de la Vision, INSERM UMRI S 968, Paris, France; ^8^Biomedical Science Tower, University of Pittsburgh, Pittsburgh, PA, United States; ^9^Robotics Institute, Carnegie Mellon University, Pittsburgh, PA, United States; ^10^Micro Air Vehicle Laboratory, Department of Control and Operations, Faculty of Aerospace Engineering, Delft University of Technology, Delft, Netherlands; ^11^The Neuroethological lab, Department of Neurobiology, The Rappaport Institute for Biomedical Research, Technion – Israel Institute of Technology, Haifa, Israel; ^12^Brain Research Center/Institute of Systems Neuroscience, National Tsing Hua University, Hsinchu, Taiwan; ^13^Laboratory of Comparative Neural Systems and Behavior, Department of Psychological and Brain Sciences, Neuroscience and Mechanical Engineering, Johns Hopkins University, Baltimore, MD, United States

**Keywords:** neuromorphic sensing, autonomous flight, bio-inspiration, flying animals, learning, flight control, energy efficiency

## Abstract

Autonomous flight for large aircraft appears to be within our reach. However, launching autonomous systems for everyday missions still requires an immense interdisciplinary research effort supported by pointed policies and funding. We believe that concerted endeavors in the fields of neuroscience, mathematics, sensor physics, robotics, and computer science are needed to address remaining crucial scientific challenges. In this paper, we argue for a bio-inspired approach to solve autonomous flying challenges, outline the frontier of sensing, data processing, and flight control within a neuromorphic paradigm, and chart directions of research needed to achieve operational capabilities comparable to those we observe in nature. One central problem of neuromorphic computing is learning. In biological systems, learning is achieved by adaptive and relativistic information acquisition characterized by near-continuous information retrieval with variable rates and sparsity. This results in both energy and computational resource savings being an inspiration for autonomous systems. We consider pertinent features of insect, bat and bird flight behavior as examples to address various vital aspects of autonomous flight. Insects exhibit sophisticated flight dynamics with comparatively reduced complexity of the brain. They represent excellent objects for the study of navigation and flight control. Bats and birds enable more complex models of attention and point to the importance of active sensing for conducting more complex missions. The implementation of neuromorphic paradigms for autonomous flight will require fundamental changes in both traditional hardware and software. We provide recommendations for sensor hardware and processing algorithm development to enable energy efficient and computationally effective flight control.

## Introduction

Autonomous flying capability in aerospace is gathering momentum with the needs for enhanced safety, sustainability, and new missions. The latest developments in data science and machine learning have accelerated the relevance of autonomy for many areas, such as space operations, unmanned aerial vehicles (UAVs), (passenger) transport, manned-unmanned teaming, and air traffic management (National Research Council [NRC], 2014; Airbus, 2020a). In future scenarios, coordination of a vast number of participants in contested air space is needed, which can be answered by autonomous and self-organizing vehicles only with unique sensing capabilities.

The latest (pilot-controlled) Airbus A350 has 50,000 sensors on board collecting 2.5 terabytes of data every day (Airbus, 2020b). Processing the amount of sensory data needed for autonomous flight by current processing technologies requires high-performance computers consuming many kilowatts of energy. In contrast, a bird has two eyes, two ears, two vestibular organs, skin, several hundreds of olfactory receptors, 25–500 taste buds, and is capable of magneto reception (Wiltscho and Wiltscho, 2019; Ornithology.com, 2020). Considering that the largest bird brains weigh 20 g, have 3 billion neurons and consume ∼0.2 W (Olkowicz et al., 2016), we need a new paradigm in bio-inspired sensors and processing, to overcome the computational and energy constraints required for autonomous operation in complex three-dimensional high-speed environments.

Neuromorphic engineering describes a large-scale system of integrated circuits that mimic neurobiological architectures present in the nervous system (Ma and Krings, 2009; Morabito et al., 2013; Zhang et al., 2020). It aims to harness the efficiencies of biological brains by developing essential computation analogs of neuronal circuits. To explore the potential of neuromorphic engineering for aviation sensors, a virtual workshop on the topic of “Neuromorphic Sensing and Processing Paradigm” was organized by Airbus, U.S. Office of Naval research Global (ONRG) and the U.S. DEVCOM Army Research Laboratory (ARL) in July 2020.

This perspective paper is one output of the workshop and aims to describe the needs, current advances and provide directions for future research related to neuromorphic sensing and processing enablers for autonomous flight missions. The goal is to call upon the scientific community and funding agencies to enable and perform the required multidisciplinary research in this area.

## Missions and Needs

Autonomous flight for passenger aircraft has the potential to deliver increased fuel savings, reduce operating costs, and allow pilots to focus on strategic decision-making and mission management rather than aircraft management. This is enabled by image-processing, decision-making support, speech processing and interpretation, and mutual human-machine trust. Urban air mobility (UAM) also requires new roles and capabilities, such as safe ground control of multiple autonomous pilot-less vehicles. Moreover, autonomy is extremely important for the next generation of defense and security air systems, where one aim is to connect manned and unmanned components in operations. Another (part of this) system can be a “swarm” of unmanned vehicles that independently coordinate to perform missions. This requires robust navigation in potentially obscure conditions, for potential missions such as reconnaissance, disaster management after catastrophes, search and rescue at sea or on ground, surveillance, automatic recognition of objects and/or people, border security, imaging, and optimized air traffic control.

General vital capabilities to achieve autonomous flight are attitude control (Goulard et al., 2016), height control and landing (Srinivasan et al., 2000; Baird et al., 2013), collision avoidance (Tammero and Dickinson, 2002; Rind et al., 2016), and navigating between places of interest (Webb, 2019). For search and rescue and security missions, high-speed vehicles able to operate in complex environments require fast response times. On the other end are long-term (>1 day) missions with a great need for low energy consumption where swarms of flying autonomous vehicles could bring benefits in terms of cost, safety, and performance. A wide range of capabilities is required, such as communication channels, predictive tracking, control of own-drone space (situational awareness), mission-dependent information, visual/spectral tracking, navigation and control. For human-autonomy teaming missions, sharing of environmental sensory information, including temperature, humidity, airflow, and olfactory inputs (smoke, biohazards, explosives, etc.) is of major importance. For space missions, autonomous navigation and situational awareness are required in, for example, space-debris collision prevention. These exquisite capabilities can be enabled by a combination of multiple centralized and distributed sensors, such as observed in biology. The multi-domain missions as described above require simultaneous sensing modalities where neuromorphic sensors may need to be combined with conventional ones, such as hyperspectral imaging.

To enable sensing and efficient real-time processing for future missions and to address real world challenges, foundational advances in neuromorphic computing are needed at the theoretical, computational, algorithmic, and hardware implementation levels to overcome today’s scalability limit of e.g. finite state machines. For example, just as insects integrate information from vision and mechanoreceptors to instantaneously correct for changes in e.g., airspeed (Buckminster Fuller et al., 2014), autonomous flying vehicles also require efficient and adaptive sensorimotor computations for instantaneous flight correction. Another challenge is navigating through complex dynamic environments with obstacle avoidance, such as flying through treetops with wind blowing through leaves and twigs. This requires luminance normalization (Hung et al., 2020), coupled with contextual processing of higher order features, environmental and task-related visual statistics, as well as feedback and recurrent processing for visual prediction. Whereas in biology such normalization, contextualization, and prediction are interdependent processes, there still exists a major gap in computational implementation today.

An ultimate task of autonomy, the ability to deal with unexpected situations, requires the system to learn and act upon sensory inputs, which is the embodiment of cognition. This requirement represents another computational challenge that may be met by neuromorphic implementation. A robust computational framework must be developed to realize learning in autonomy that mimics, to some degree, even simple animals. The inability to provide training data for all scenarios poses a challenge for full autonomous flight to meet commercial, defense, security and space mission needs of the future. Given the vast area of research in neuromorphic processing, we only treat learning and decision-making in the context of sensing here in our discussion.

## Current Advances in Neuromorphic Sensing

Neuromorphic sensing occurs through a device that perceives changes in a certain parameter and outputs them into a stream of events (“spikes”) (Morabito et al., 2013). Thus, essentially any sensing modality can be converted into an event-based (EB) detector. For example, pixels in EB cameras can signal brightness changes exceeding some threshold; microphones in EB audio sensors can react to sound intensity variations in certain frequency ranges (Li et al., 2012; Liu et al., 2014; Inilabs.com, 2020); EB chemical or olfactory sensor arrays or bio-hybrid sensors can fire as a result of chemical element concentration deviations (Koickal et al., 2007; Chiu and Tang, 2013; Vanarse et al., 2017; Anderson et al., 2020); EB tactile sensors respond to changes in force and movement (Kim et al., 2018; Baghaei Naeini et al., 2020).

The most prevalent example of EB sensors are video cameras (Lichtsteiner et al., 2008; Posch et al., 2011; Chen et al., 2012; Brandli et al., 2014; Son et al., 2017; Taverni et al., 2018; Clarke, 2020), which despite their relatively recent birth have already experienced significant evolution. Event based cameras have the necessary properties to become the foundational sensors for autonomous flight, solving the challenges of power, computing, and timing requirements and enabling local decision-making. In addition to being affordable and fast, they offer independence from lighting conditions and a large dynamic range. Event based sensors can process the equivalent of several hundred kHz frames using conventional adaptive CPU hardware for computations that are impossible to carry out with classic frame-based sensors. Examples are: real-time optical flow (Benosman et al., 2014), pose estimation (Reverter Valeiras et al., 2016, 2018), time-based machine learning (Lagorce et al., 2017), aperture free optical flow (Akolkar et al., 2020), and many other applications that have been now developed using pure temporal mechanisms performed at almost real-time speeds.

Another neuromorphic approach to active sensing, includes the world’s first spiking neural network-based chip that was announced recently for radar signal processing. The first application was reported to encompass the creation of a low-power smart anti-collision radar system for drone collision avoidance; future plans are to process a variety of active sensor data including electrocardiogram, speech, sonar, radar and LIDAR streams (Liu, 2020). Reportedly, the chip consumes 100 times less power than traditional implementations and provides 10X reduction in latency.

The algorithmic and hardware transitions to EB sensing platforms are driven by the desire to reduce latency, to achieve orders of magnitude improvement in energy efficiency, dynamic range, and sensitivity, to solve complex control problems with limited computing resources and to attain autonomous system’s capability of adapting to operation in unpredictable dynamic environments. Hence recently, they have been applied successfully in space surveillance applications (Roffe et al., 2021) and for controlled landing of micro-air vehicles (Dupeyroux et al., 2020). However, despite the progress achieved in the last decade by state-of-the-art neuromorphic sensors, there are several fundamental barriers separating them from real life applications. For example, visual EB sensors have limited ability to handle high focal plane array utilization due to complex illumination or clutter as well as pixel response inhomogeneity. In terms of sensor data processing, a major current challenge is to develop spike neural network learning principles, concurrently advancing both the algorithms and hardware, to enable the disparate sensor data fusion inherent to biological sensing.

## Bio-Inspiration From Flying Animals and Insects

To address the challenges of energy-efficient real-time processing of multiple sensing modalities, and the ability to deal with unexpected situations as described above, we can look toward flying animals and insects. For example, spatial navigation builds upon a network of cognitive functions. Animals that rely on active sensing present particularly powerful models to guide the implementation of cognitive functions in the design of autonomous navigation systems, as their actions reflect cognitive states and directly influence signals used to represent the environment, which, in turn, guide 3D movement. Echolocating bats, for example, transmit sonar cries, and use information carried by returning echoes to determine the 3D position, size, and other features of objects in the environment (Simmons and Vernon, 1971; Simmons, 1973; Griffin, 1974; Busnel and Fish, 1980; Nachtigall and Moore, 1988; Moss and Schnitzler, 1989; Thomas et al., 2004; Moss and Surlykke, 2010). Central to successful 3D navigation of autonomous vehicles in cluttered environments is spatial attention. Attention invokes mechanisms that allocate computational resources to selectively process and enhance relevant information from the environment (Broadbent, 1957). It has been demonstrated in bats that sonarguided attention drives state-dependent processing of echo returns in brain regions that play key roles in spatial navigation. Specifically, attention invokes the sharpening of auditory midbrain neurons that encode an object’s 3D location in egocentric coordinates and hippocampal place cells that encode an animal’s 3D location in allocentric coordinates (Yartsev and Ulanovsky, 2013; Kothari et al., 2018; Wohlgemuth et al., 2018). Moreover, bats adjust the directional aim and temporal patterning of echolocation signals to inspect objects in the environment, which reveals adaptive sonar signal design, tailored to the task at hand. Current sonar technologies do not yet implement adaptive signal transmission and processing, which may explain superior performance of animals over artificial devices. New technologies that incorporate more complete knowledge of animal echolocation systems pave the way for advanced 3D sonar-guided navigation of autonomous vehicles in dark and cluttered environments.

For barn owls, as efficient predators adapted for hunting rodents in extremely low light conditions, attention is also of key importance to prevent overload of information processing capacity and for stable behavioral control in face of multiple distractors in cluttered and noisy environments (Posner, 2016). Neurons in the optic tectum of barn owls respond preferentially to visual and auditory stimuli that break a regular pattern of their background (Zahar et al., 2018) or that are categorically stronger from competing stimuli (Mysore et al., 2011). Unique neural adaptation (Netser et al., 2011), circuit motifs (Mysore and Knudsen, 2012) and top down modulation (Winkowski and Knudsen, 2008) that facilitate the stimulus selection process at the neuronal level have been identified. Such insights on the mechanisms of the barn owl’s neural activity may teach us information-processing strategies that are efficient and behaviorally useful.

The barn owl’s intriguing capability to avoid obstacles in dark conditions seems to rely on spatial memory and strong sense of self position in the memorized map of space (Payne, 1971). Preliminary results have begun to reveal the neural representation of the owl’s location and direction in space and provide a promising avenue for new inspirations about autonomous aviation in extreme light conditions (Agarwal and Gutfreund, 2019).

Another entry point is to focus on insect brains that are known to be of lesser complexity and therefore more adapted to modeling. Insect brains are particularly adapted to resource-constrained scenarios as in the case of flying machines while showing amazing functionalities that allow them to perform complicated tasks with great ease such as (visual) collision avoidance, localization, communication, navigation, odor source localization and social interaction in unknown unpredictable environments (Smith, 2007; Schneider and Levine, 2014; Weir and Dickinson, 2015; Fu et al., 2019; Huang et al., 2019; Hu et al., 2020).

For example, *Drosophila melanogaster*, or fruit fly, has a small brain with ∼100,000 neurons that are highly efficient in sensory processing. Although having an extremely poor spatial resolution (only ∼800 pixels in each compound eye), the visual system of fruit flies and other type of flies are highly sensitive to movements (Borst et al., 2010; Borst, 2018), inspiring the development of EB cameras described earlier. Moreover, there are several downstream layers of neural circuits that compute and process optical flow (Weir and Dickinson, 2015; Mauss and Borst, 2020). This innate neural “computation” endows a fly with the abilities to detect threats (high-speed moving objects), avoid obstacles, control its flight course (Mauss and Borst, 2020), and estimate its orientation (Su et al., 2017), which is exactly what we need for our autonomous flying vehicles, without the need for more than rudimentary object recognition and classification, which are computationally expensive in today’s artificial neural network architectures. Other examples are represented by locusts and flies that can detect visual collisions by a special neural structure (Fu et al., 2019). Recently, it has been modified into bio-plausible neural models that were applied on autonomous mobile robots and also UAVs with constrained computational resources (Huang et al., 2019; Hu et al., 2020).

## Outlook Toward Advancing Neuromorphic Applications

So, how can we translate nature’s amazing capabilities into autonomous flying vehicles with limited energy supply? For example, flying nano-drones (Ma et al., 2013; Karásek et al., 2018) mimicking capabilities of fruit flies will unlock novel opportunities, such as development of nano-drone swarms that can explore and monitor unknown indoor or outdoor environments (Brambilla et al., 2013; McGuire et al., 2019). Recently, a neuromorphic chip was used in the control loop of a flying drone able to perform smooth optical flow landings, like honeybees, which immediately illustrated the energy efficiency and speed promised for such neuromorphic applications (Dupeyroux et al., 2020). Computational principles of dynamic vision of fruit flies or other insects can be implemented together with EB cameras, and used in parallel with the slower and energetically demanding computer vision systems that are designed for object recognition. This way a UAV can detect obstacles or potential threats even before they are recognized while using low-powered passive vision. For larger aircraft, advances in neuromorphic computing could lead to improved sensing and sensory fusion, including real-world resilience and prediction, inspired by the role of biological recurrent networks in solving such challenges (Tang et al., 2018; Kubilius et al., 2019).

Autonomous flight with neuromorphic efficiency requires a cross-disciplinary effort, and EB temporal computation requires new thinking. One missing piece of the puzzle to create truly neuromorphic systems is the computational hardware. It is expected that such architecture will be extremely low power while allowing to truly operate in real-time at the native resolution of the sensor. An optimal solution would be to approach the problem by considering the timing of events and developing techniques where each event incrementally adds information to what has already been computed (Benosman et al., 2014). This idea of local computation is not new and has been described to be present in most real neural networks where the layers of neurons process information at their own independent time scales based on the received sensory data rather than relying upon any form of “global” clock or memory.

Potential advantages of neuromorphic computing for active vs. passive imaging (e.g., bat echo-location vs. owl vision) should also be explored. Active vision (e.g., dense LIDAR) can provide limited 3D sensing but is challenged by objects such as trees, poles, and traffic lights (Chauhan et al., 2014), whereas passive imaging is preferred for maintaining stealth but is energetically expensive. Both areas of research have been dominated by convolutional approaches, and an open question is how to fuse active and passive sensors, including antennae, and their data for efficient, resilient, and adaptive sensory-decision-motor loops. Sensory information in biological brains is represented in the same way for all signals, there is occurrence and temporal correlation, no distinction between inputs, and a generic way of generating events triggered by the data. This saves a lot of time and energy and EB sensors could aid in emulating biology in this sense.

Top-down (internal guidance – mission driven) and bottom-up (externally driven) attention (Katsuki and Constantinidis, 2014) are the neural processes that may solve the bottleneck issue in sensory information processing. With these two types of attention mechanisms, our brain’s central executive unit is able to focus on mission-relevant sensory signals while maintaining flexibility in rapidly switching to other sensory signals that occur unexpectedly. Predictive coding might play a crucial role here because it generates and updates an internal representation (or mental model) of the environment, and attention is required only when a prediction error occurs, which causes the system to shift to a high-power mode.

An additional layer of complexity is presented by neuromorphic computing inspired by biological principles for learning, which is needed for adaptive, resilient, and resource-efficient distributed sensing and learning (i.e., by swarms and other sensors) (Thomas, 1997; Abdelzaher et al., 2018), e.g., of target signatures and harsh environmental conditions, and for assured low-bandwidth communication. Progress on these challenges would create a framework of foundational principles, e.g., for predicting patterns and decisions from complex dynamics.

**FIGURE 1:**
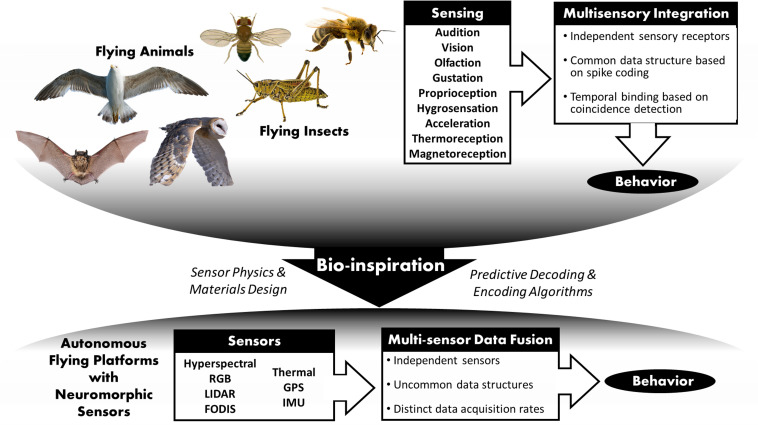
Schematic route from bio-inspired behaviors toward neuromorphic sensors for autonomous flight. Animal figures are all covered by copyright with Creative Commons through https://www.pexels.com.

We have identified a clear need to enhance understanding of neurosensory systems in nature’s flying creatures, which shall result in new and better mathematical models needed for autonomous flying vehicles, see Figure 1. The long-term goal is hardware and software design and prototyping for interacting autonomous vehicles. Our target is neuromorphic hardware that aims at mimicking the functions of neural cells in custom synthetic hardware that is analog, digital, and asynchronous in its nature of information processing and is vastly more energy-efficient and lighter than classical silicon circuitry. It is expected that such a neuromorphic technology will disrupt existing solutions and be a key enabler for real-time processing of different sensor modalities by lower cost, lower energy consumption, lower weight, adaptable to changing missions, while providing enhanced and resilient performance and saving human lives.

In Table 1, we have created an overview of the current challenges toward autonomous flight and how the biology of flying creatures can inspire us in the coming years to reach the desired milestones. To summarize our recommendations:

**TABLE 1:** Neuromorphic sensing for autonomous capabilities – roadmap.

**Desired functions**	**Current challenges**	**Bio-inspiration**	**Estimated timeline (years)**

High-speed in complex environment (obstacle and collision avoidance, mission performance)	Dynamic range and sensitivity, response times, sensory fusion, multiple agents in congested space	Fruit fly innate flight control and survival capabilities, swarming	5 (UAM) – 20 (combat systems)

Robust navigation	Obscuration and glare conditions, GPS denied environment	Visual and magnetoreceptive capabilities of flying animals, spatial memory	5–10

Increasing complexity of flight control, air/ground transition efficiency	Sensorimotor integration, translation between small and large platforms and different degrees of complexity	Resilience to wind gusts, innate landing/perching/take-off, differences in brain processing between insects and birds	5–10

Computing and sensing efficiency	Scalability, power, weight	Resource-limitation in biology (few neurons in small low-weight brain), sensory fusion	10–15

Multi-sensory awareness	Sensory fusion and energy consumption, reliable automated object recognition	No distinction between neurons signalling in different sensory processing systems, learning, attention and recognition	5–15

Cognition/adaptability	Deep understanding of brain learning mechanism	Learning, attention, decision and reward systems	30+

*Please note that the mentioned challenges involve a mix of both algorithms and hardware.*

1. Develop EB neuromorphic sensor hardware and processing algorithms to support resilient and efficient navigation and collision avoidance
2. Develop computationally efficient flight control with fast sensor-to-actuator responses to support resilience
3. Develop neuromorphic attentional, sensory fusion, and representational mechanisms to increase efficiency and goal-directed performance in complex scenarios
4. Develop neuromorphic approaches to learning for adaptive and predictive sensing and control
5. Develop principles to integrate neuromorphic and convolutional approaches to harness their mutual advantages

## Data Availability Statement

The original contributions presented in the study are included in the article, further inquiries can be directed to the corresponding author/s.

## Author Contributions

PP created the outline and wrote introduction, collected inputs, and integrated them in the full manuscript. AK and CH wrote sections and reviewed manuscript. YG, C-CL, RB, CM, and GC reviewed the manuscript and contributed sections that were integrated in the manuscript by PP. AS and FG reviewed the manuscript. All authors contributed to the article and approved the submitted version.

## Disclaimer

The views and conclusions contained in this document are those of the authors and should not be interpreted as representing the official policies, either expressed or implied, of the DEVCOM Army Research Laboratory or the U.S. Government. The U.S. Government is authorized to reproduce and distribute reprints for Government purposes notwithstanding any copyright notation herein.

## Conflict of Interest

PP and AS are employed by the company Airbus Defence and Space GmbH. The remaining authors declare that the research was conducted in the absence of any commercial or financial relationships that could be construed as a potential conflict of interest.
